# Multicenter Analysis of Treatment Outcomes for Systemic Therapy in Well Differentiated Grade 3 Neuroendocrine Tumors (NET G3)

**DOI:** 10.3390/cancers13081936

**Published:** 2021-04-16

**Authors:** Leonidas Apostolidis, Arianna Dal Buono, Elettra Merola, Henning Jann, Dirk Jäger, Bertram Wiedenmann, Eva Caroline Winkler, Marianne Pavel

**Affiliations:** 1 National Center for Tumor Diseases (NCT) Heidelberg, Department of Medical Oncology, Heidelberg University Hospital, 69120 Heidelberg, Germany; dirk.jaeger@med.uni-heidelberg.de (D.J.); eva.winkler@med.uni-heidelberg.de (E.C.W.); 2 Department of Gastroenterology and Hepatology, Charité University Medicine, 13353 Berlin, Germany; arianna.dalbuono@humanitas.it (A.D.B.); elettra.merola@apss.tn.it (E.M.); henning.jann@charite.de (H.J.); bertram.wiedenmann@charite.de (B.W.); marianne.pavel@uk-erlangen.de (M.P.); 3 Department of Medicine 1, Division of Endocrinology, Friedrich Alexander University Erlangen-Nuremberg, 91054 Erlangen, Germany

**Keywords:** NET G3, neuroendocrine tumor, neuroendocrine carcinoma, systemic therapy, chemotherapy, peptide receptor radionuclide therapy, first-line

## Abstract

**Simple Summary:**

Neuroendocrine tumors grade 3 (NET G3) are a newly defined subgroup of neuroendocrine neoplasms. They do not respond well to platinum + etoposide-based chemotherapy. The alternative suggested options have not been analyzed in untreated NET G3 patients so far, therefore the optimal treatment strategy for these tumors is unclear. In our analysis we showed that FOLFOX is the most active regimen holding the highest chance of tumor shrinkage, whereas temozolomide/capecitabine is the most effective, leading to the most durable tumor control.

**Abstract:**

Well-differentiated grade 3 neuroendocrine tumors (NET G3) have been distinguished from poorly differentiated neuroendocrine carcinomas (NEC) in the most current WHO classifications. Commonly applied first-line chemotherapy protocols with cisplatin or carboplatin in combination with etoposide (PE) are less effective in NET G3 than NEC. Suggested alternative treatment protocols have not been studied in first-line therapy of NET G3 so far. We performed a retrospective analysis of patients with NET G3 in the databases of 3 German cancer centers. Out of 142 patients, 136 patients received palliative first-line therapy: overall response rate (ORR) was 35.1% for PE (*n* = 37), 56.4% for FOLFOX (*n* = 39), 27.3% for temozolomide/capecitabine (TEM/CAP) (*n* = 22), 45.0% for streptozotocin/5-fluorouracil (STZ/5-FU) (*n* = 20), and 16.7% for other (*n* = 18). Median progression-free survival (PFS) for PE was 6.9 months. Compared to PE, PFS in the other treatment groups was 6.9 months for FOLFOX (*p* = 0.333), 12.0 months for TEM/CAP (*p* = 0.093), 4.8 months for STZ/5-FU (*p* = 0.919), and 14.1 months for other (*p* = 0.014). In a univariate setting, all non-PE patients combined showed a significantly prolonged PFS vs. PE (9.0 months; *p* = 0.049) which could not be confirmed in a multivariate analysis. In conclusion, NET G3 with FOLFOX showed the highest ORR, and with TEM/CAP showed the longest PFS. Further prospective evaluation of the optimal therapeutic strategy for this tumor entity is needed.

## 1. Introduction

In the WHO classification from 2010, all neuroendocrine neoplasms (NEN) with a proliferation rate (Ki67) > 20% had been classified as poorly differentiated grade 3 neuroendocrine carcinomas (NEC) in contrast to well differentiated grade 1 and 2 neuroendocrine tumors (NET G1/G2) [1]. However, further analysis of clinical characteristics in this group revealed a subgroup with a biologically heterogeneous behavior. Compared to the highly proliferative aggressive small cell and large cell NEC, the histology was characterized by a morphologically good differentiation, the Ki67 was mainly below 55%, and the overall survival of patients was more favorable. This led to the definition of well differentiated grade 3 neuroendocrine tumors (NET G3) in the most recent WHO classifications from 2017 and 2019 [2,3]. Accordingly, NET G3 are defined as morphologically well differentiated NEN with a Ki67 > 20%. Retrospective data suggest that commonly applied first-line chemotherapy protocols with cisplatin or carboplatin in combination with etoposide (PE) are less effective in NET G3 than in NEC [4,5,6,7].

Therefore, current treatment guidelines suggest alternative first-line treatment protocols like temozolomide + capecitabine (TEM/CAP), streptozotocin + 5-fluorouracil (STZ/5-FU), and oxaliplatin + 5-fluorouracile (FOLFOX) [8,9,10]. However, until now, those treatments have only been evaluated retrospectively in pretreated grade 3 NEN, and data for efficacy of those treatments in first-line setting of untreated NET G3 are lacking [11,12].

The aim of this multicenter analysis was to evaluate treatment outcomes for NET G3 with a focus on the efficacy of different first-line regimens.

## 2. Materials and Methods

We performed a retrospective analysis of all patients diagnosed with NET G3 in the NEN databases of 3 German cancer centers treated between August 2010 and August 2020. Patients with a history of previous NET G1/G2 and new diagnosis of NET G3 were also included, irrespective of their previous treatment. All histopathological reports were reviewed by investigators with >10 years of experience in diagnosis and treatment of NEN in order to comply with the most current WHO classifications of 2017 and 2019. Criteria for diagnosis of a NET G3 were Ki67 > 20% with a well differentiated organoid or nesting morphology. Patients with poorly differentiated large cell or small cell morphology were excluded, as were ambiguous cases and mixed neuroendocrine non-neuroendocrine neoplasms (MiNEN). A local NEN experienced pathologist was consulted in case the report was lacking information or was unclear. All cases were discussed in the multidisciplinary tumor boards of the respective institutions.

Patient characteristics like demographics, tumor stage, proliferation rate (Ki67), functional activity, and tumor markers were collected at the timepoint of NET G3 diagnosis. The response according to RECIST 1.1 criteria was recorded. Indication for initiation of systemic therapy in every treatment line was tumor progression. Progression-free survival (PFS) was defined as the time span between the start of the respective therapy and the date of progression or death due to any cause. Overall survival (OS) was defined as the time length between diagnosis of NET G3 and the date of death from any cause. For subgroup comparison of systemic treatments, patient characteristics were collected at the timepoint of initiation of the respective treatment.

Statistical analysis was carried out using SPSS™ for Windows™ Software v25.0 (SPSS, Chicago, IL, USA). The distribution of continuous variables was presented as median and range. Comparisons between more than two subgroups were performed with the Kruskal-Wallis test for continuous variables and with the χ^2^ test for non-continuous variables. For two subgroups, comparisons were performed with the Man-Whitney U test for continuous variables and with Fisher’s exact test for non-continuous variables. Survival analysis was calculated using the Kaplan-Meier method, and differences in survival were analyzed using the log-rank test. Risk factor analysis was developed following the Cox-regression hazard models. Results were expressed as hazard ratio (HR) and 95% confidence interval (CI). A *p*-value of <0.05 was considered significant. Median follow-up was calculated using the reverse Kaplan-Meier method.

The trial was performed according to World Medical Association Declaration of Helsinki and approved by the respective institutional research ethics committee (approvals S-207/2005, S-428/2014, and EA2/064/09).

## 3. Results

### 3.1. Patient Characteristics, Survival, and Follow-Up

A total of 142 patients could be identified (Table 1). Median Ki67 was 30% and primary tumors were located in the pancreas in 69.7% of cases. Sixteen patients showed endocrine functional activity (carcinoid syndrome *n* = 10, insulinoma *n* = 3, glucagonoma *n* = 2, gastrinoma *n* = 1, ectopic adrenocorticotropic hormone production *n* = 1).

Twenty-three patients had a history of prior NET G1/G2 diagnosis with a median latency up to the NET G3 diagnosis of 49.0 months (range 6.9–304.0). Those patients (pancreatic *n* = 13, small intestinal *n* = 7, colorectal *n* = 1, stomach *n* = 1, unknown *n* = 1) had been treated for their NET G1/G2 with a median of 2 lines of systemic therapy (range 0–6), most commonly somatostatin analogues (SSAs) (*n* = 14), peptide receptor radionuclide therapy (PRRT) (*n* = 11), chemotherapy (*n* = 6), and targeted therapy (*n* = 6).

**Figure 1:**
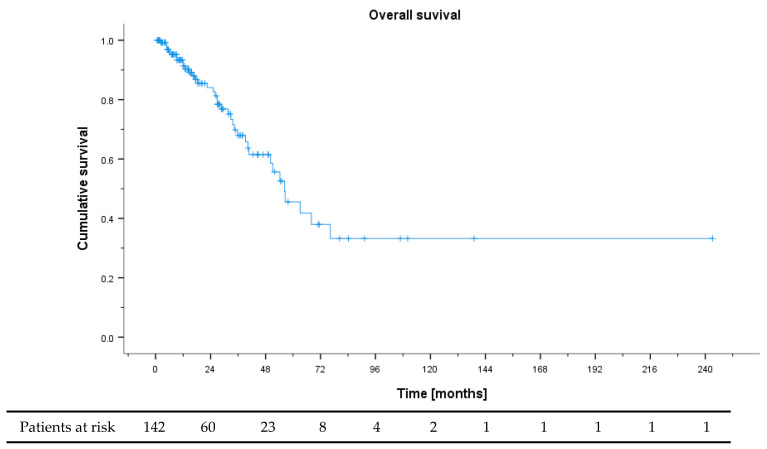
Overall survival of all patients from NET G3 diagnosis.

Median overall survival (OS) of the whole cohort was 56.3 months (95% CI 43.7–68.9) with a median follow up of 31.9 months (Figure 1). Resection of primary tumor and metastasis was performed in 40.1% and 17.6% of patients, respectively.

### 3.2. First-Line Therapy

One hundred and thirty-six patients received palliative first-line therapy (the remaining 6 had received only curative primary resection with no need of systemic therapy): the most common treatments were PE (*n* = 37), FOLFOX (*n* = 39), TEM/CAP (*n* = 22), and STZ/5-FU (*n* = 20). The remaining 18 patients received somatostatin analogues (*n* = 6), peptide receptor radionuclide therapy (PRRT) (*n* = 3), other chemotherapeutic regimens (*n* = 2), sunitinib (*n* = 3), everolimus (*n* = 1), and multimodal combination approaches of systemic therapy and tumor debulking (*n* = 3). Those patients were subsumed in the group “other”.

**Figure 2:**
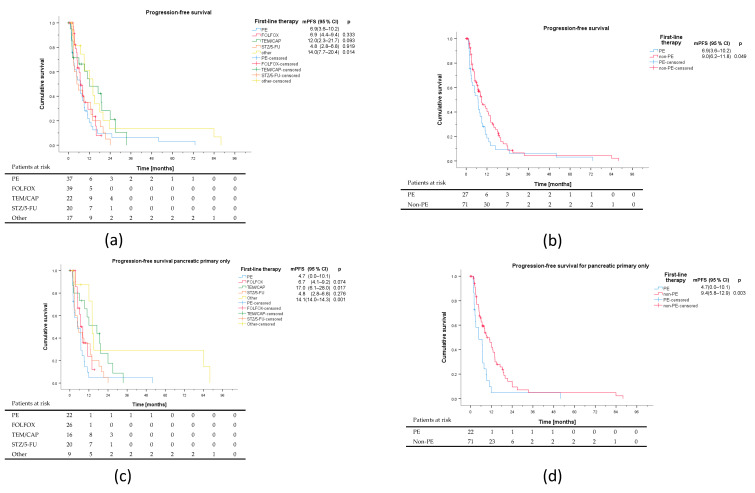
Progression-free survival of different first-line regimens. Patients receiving SSAs, PRRT, other chemotherapeutic regimens, targeted agents and multimodal combination approaches were subsumed under other. Median PFS is listed with 95% confidence intervals. All *p* values are delineated in comparison to platinum + etoposide (PE). (**a**) Comparison of the main treatment groups. (**b**) Comparison of PE vs. non-PE. (**c**) Comparison of the main treatment groups for pancreatic primary only. (**d**) Comparison of PE vs. non-PE for pancreatic primary only.

Overall response rate (ORR) and disease control rate (DCR) were highest for FOLFOX with 52.8 and 80.6%, respectively (Table 2), with the differences in ORR reaching statistical significance (*p* = 0.032). Median progression free survival (PFS) for PE was 6.9 months (Figure 2A). Compared to PE, PFS was numerically longest for TEM/CAP with 12.0 months (*p* = 0.093) and for “other” with 14.1 months (*p* = 0.014). PFS for FOLFOX and STZ/5-FU were 6.9 (*p* = 0.333, compared to PE) and 4.8 months (*p* = 0.919), respectively. All non-PE patients combined showed a significantly prolonged PFS vs. PE (9.0 vs. 5.2 months, *p* = 0.049) (Figure 2B).

The 3 patients with multimodal highly individualized treatment in the group “other” showed an exceptionally long time to progression: the first patient with a pancreatic NET G3, Ki67 25%, was diagnosed at the age of 14 and received a combination therapy of temozolomide and thalidomide, followed by intraarterial PRRT and maintenance treatment with sunitinib, and she progressed after 81.9 months with new brain metastases. The second patient with a pancreatic NET G3, Ki67 30%, got extensive tumor debulking, followed by octreotide and PRRT, and he progressed after 88.1 months with new liver and lymph node metastases. The third patient underwent resection for nodal positive breast cancer, postoperative staging revealed a borderline-resectable pancreatic lesion, so a chemotherapy with gemcitabine and nab-paclitaxel was performed in adjuvant intention for breast cancer and neoadjuvant intention for suspected pancreatic cancer. Resection of the pancreatic lesion after chemotherapy revealed a NET G3, Ki67 25%. Subsequent DOTATOC-PET/CT showed several SSTR positive bone lesions which in conjunction with negative bone scintigraphy were attributed to the NET G3. Those lesions were followed-up without any further NET-specific treatment under adjuvant endocrine therapy with letrozole for breast cancer and were progression-free at 17.3 months ongoing at time of analysis.

When comparing the different treatment groups, some remarkable differences with respect to baseline features were notable (Table 3): The PE group showed the highest median Ki67, and the lowest percentage of somatostatin receptor (SSTR) positive tumors. The FOLFOX group had a higher Ki67, a higher proportion of patients with elevated LDH, also less SSTR positive tumors, and the highest proportion of patients with prior NET G1/G2 history. Gastric, small intestinal, and colorectal primaries were mainly found in the PE and FOLFOX group, while the STZ/5-FU group had only pancreatic primaries, and also the lowest number of patients with elevated NSE. Differences in Ki67, primary localization, age, prior NET G1/G2 history, and metastatic pattern reached statistical significance. When looking only at patients with pancreatic primary, PFS for PE, FOLFOX, TEM/CAP, STZ/5-FU, and others were 7.6, 8.5 (*p* = 0.074), 15.2 (*p* = 0.017), 8.8 (*p* = 0.276), and 33.5 months (*p* = 0.001) respectively (Figure 2C). PFS of non-PE combined was 14.5 months (*p* = 0.003) (Figure 2D). For the smaller group of non-pancreatic primaries, no significant differences regarding PFS could be detected, neither between the five treatment groups nor between PE and non-PE regimens (Supplementary Figure S1).

In a univariate analysis for PFS risk factors, only the choice of PE vs. non-PE regimen could be identified as a significant variable (Table 4). On the multivariate level using parameters from the univariate analysis with a statistical trend of *p* < 0.25, no significant risk factors could be detected. An additional multivariate analysis with only non-PE vs. PE regimen and Ki67 as covariates, excluding the 3 multimodally treated patients with exceptionally long PFS, also showed no significant risk factors (Supplementary Table S1).

### 3.3. Second-Line Therapy and Beyond

**Figure 3:**
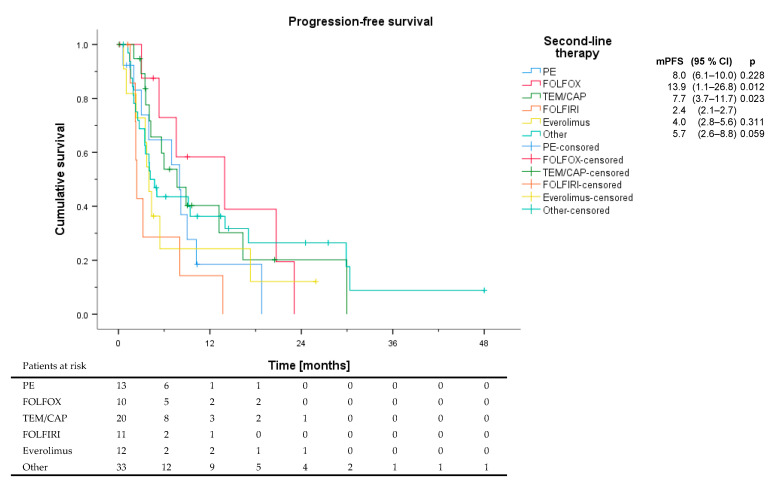
Progression-free survival of different second-line regimens. Patients receiving PRRT, SSAs, targeted agents, checkpoint inhibitors, and other chemotherapy regimens like STZ/5-FU and topotecan were subsumed under other. All *p* values are delineated in comparison to FOLFIRI.

Ninety-nine patients received second-line systemic therapy with a median PFS of 5.6 months (Figure 3). The most frequent treatments were PE (*n* = 13), FOLFOX (*n* = 10), TEM/CAP (*n* = 20), FOLFIRI (*n* = 11), and everolimus (*n* = 12). The remaining 33 patients received a multitude of other treatments, each with *n* < 8, including PRRT (*n* = 7), SSAs (*n* = 5), targeted agents (*n* = 2), checkpoint inhibitors (*n* = 7), and other chemotherapy regimens (STZ/5-FU, *n* = 5, topotecan, *n* = 4). In second-line, patients receiving FOLFIRI showed the shortest PFS, significantly shorter than with FOLFOX or TEM/CAP. Both the highest ORR and DCR as well as the longest PFS could be observed for the FOLFOX group (Table 5). In the other groups, 6 responses were noted in 3 patients receiving PRRT, 1 patient receiving STZ/5-FU, and 2 patients with multimodal treatment. Of 7 patients receiving second-line treatment with an immune checkpoint inhibitor (all as monotherapy), no responses were detected. Looking at the features of second-line treated patients, while the median Ki67 in the 10 patients of the second-line FOLFOX group was 30%, only 5 patients had a pancreatic primary, and 50% had been refractory to first-line treatment (Supplementary Table S2).

Third- and fourth-line treatment was administered in 66 and 38 patients, respectively. The median number of treatment lines in patients receiving systemic therapy was 3 (Range 1–9).

## 4. Discussion

This study is the first comprehensive multicenter analysis for NET G3 with a focus on different treatment strategies and clinical outcomes. Of the distinctly defined protocols used in first-line, FOLFOX showed the highest ORR, and TEM/CAP showed the longest PFS. However, several tumor features have probably impacted treatment choices, since significant differences in the treatment groups could be noted, suggesting that patients with a more aggressive disease course with higher Ki67, elevated LDH, and SSTR negative tumors received FOLFOX, whereas TEM/CAP patients might have had a more indolent disease. On the other hand, upon analysis of risk factors for progression, only the use of PE vs. non-PE chemotherapy could be identified as significant at univariate level. This signal for better outcomes for non-PE regimens could not be confirmed in the multivariate analysis.

The patient characteristics of our study are in line with previous reports for NET G3 [5,6,7]. Although NET G3 are most commonly diagnosed in the pancreas, other extrapancreatic primaries could be detected in one third of the cases. We could also observe transition of previously diagnosed NET G1/G2 into NET G3 in 16.3% of patients. For pancreatic NEN G3, differences between NET G3 and NEC have been established on a molecular level: similar to NET G1/G2, pancreatic NET G3 harbor alterations in the MEN1, DAXX/ATRX and mTOR genes, on the other hand, TP53 and RB1 mutations are almost exclusively detected in NEC and not in NET G3 [2,3,13,14]. This emphasizes the hypothesis of a biological continuum from NET G1/G2 to NET G3.

When comparing our results to other studies, one main issue is the introduction of the new WHO classifications of 2017 and 2019 which for the first time officially defined the tumor entity of NET G3. In previously published works, when the WHO 2010 classification was still in place, NET G3 are often not explicitly mentioned. However, when these works especially refer to the NEN subgroup with Ki67 20–55% it can be assumed that a substantial proportion of NET G3 were included. Therefore, taking into consideration the limited NET G3 evidence in general, it is important to discuss our results also in context with these older studies, even if the exact number of NET G3 patients in these analyses is unclear.

Based on the data of the NORDIC NEC study and several subsequent reports, PE is considered an inferior treatment regimen for NET G3 [4,5,6,7], and therefore not recommended as first-line treatment in current guidelines [8,9,10]. The low efficacy of PE was even one of the main observations which impacted the need for a revised classification of NEN, and lead to the definition of the NET G3 category. Our data are consistent with these findings, showing that PE seemed inferior to many of the other applied protocols. However, also in the PE group one third of the patients had a tumor response, while the highest median Ki67 was observed in this group. Also used as a second line, although only in a small number of patients, ORR was still above 40% with PE.

FOLFOX has shown effect in multiple treatment settings for NEN [11,15,16]. In a retrospective analysis of pretreated NEC G3, FOLFOX in second line after failure of standard PE chemotherapy showed activity in 30% of patients; the median PFS was more favorable for patients with Ki67 < 55% (6.2 months) versus Ki67 > 55% (3.6 months), respectively [11]. Although patient populations of low-grade NEC are not comparable to NET G3, the reported PFS findings are in line with our results. In another series median PFS was 12 months in NET G3 vs. 8 months in NEC [15]. FOLFOX showed an exceptionally high response rate in our study in first-line, and even was the most effective treatment regarding ORR and PFS in second-line. However, the patient number in the second-line group was very small and the favorable results could be explained by an enrichment of non-pancreatic primaries as well as first-line refractory patients in this group.

TEM/CAP has been widely studied in well differentiated pancreatic NET [17,18]. The data in NEN G3 is limited but TEM/CAP was one of the first treatments showing efficacy in NEC G3 as a second-line therapy [12]. In that study, efficacy of TEM/CAP was higher in the subgroup of patients with a Ki67 < 55%, although ORR and PFS of this subgroup was not explicitly reported. Based on the patient and tumor features (high percentage of SSTR expression, high proportion of patients with CgA elevation, favorable OS) reported in that study, it can be assumed that a large proportion of the studied population reflect rather NET G3 instead of NEC. Of note, at the time of the study the current WHO classification was not yet in place. Subsequent works with TEM/CAP have also included small numbers of NET G3 without reporting the specific outcome of this group [19] or showed promising responses in the subgroup of Ki67 20–55% without explicitly referring to NET G3 [20]. Our study is the first to show promising efficacy of TEM/CAP in 22 patients with untreated NET G3 regarding ORR, DCR, and PFS.

STZ/5-FU is well established for pancreatic NET G1/G2 [10,21,22]. However, although considered as one of the potential first-line treatment options for NET G3 [8], there are no structured clinical data evaluating its efficacy in this setting. Our data are the first to show considerable efficacy of STZ/5-FU in pancreatic NET G3 with a high ORR and DCR, although PFS seems lower than for TEM/CAP.

FOLFIRI has been studied in pretreated NEC [23]. In our study in NET G3, FOLFIRI was mainly used as second-line option and had only marginal efficacy. The number of patients was low, however, with only 1 of 11 patients showing objective response.

The mTOR inhibitor everolimus has been established as a standard of care treatment for NET G1/G2 in several phase III trials [24,25]. Results of a phase II study in NEC G3 in a Japanese population showed only very limited activity of everolimus [26]. There was no difference observed for PFS between Ki67 > 55% and < 55%, with data of NET G3 not being reported. In a retrospective series of 15 patients with pancreatic NEC (median Ki67 30%) median PFS was 6 months, however 40% had disease stabilization for at least 12 months [27]. In our small series of 12 patients who received everolimus as second-line therapy, only 2 patients developed partial response and median PFS in the overall cohort was short. Data from prospective studies in NET G3 are pending to elucidate further the value of everolimus in this setting.

The tyrosine kinase inhibitor sunitinib is approved for pancreatic NET G1/G2 [28]. Retrospective data suggest some activity of sunitinib in individual patients with NEN G3 [29]. Since only very few patients were treated with sunitinib in our analysis, no final conclusions regarding this treatment could be drawn. Prospective studies are ongoing with novel tyrosine kinase inhibitors, such as cabozantinib in NET G3 and low-grade NEC.

PRRT has shown promising activity in SSTR positive NEN G3 in several retrospective studies with median PFS ranging from 9–23 months [30,31,32,33]. The response was more durable if Ki67 was lower than 55%. In one of the studies, median PFS was 6.8 months in patients with Ki67 > 35%. In the other studies, median PFS ranged between 4 and 6 months if Ki67 was above 55%. Although only few patients had received PRRT in our analysis, some responses could be observed, and the proportion of SSTR positive patients as potential candidates for PRRT was quite high.

Considering novel treatment approaches like immunotherapy, 6 patients receiving immune checkpoint blockade as monotherapy in second-line showed no objective responses. This is in line with previous studies, showing immune checkpoint monotherapy only marginally effective in NEN G3 [34]. Recently, encouraging responses in grade 3 NEN patients in two phase II studies with combined immune checkpoint blockade with ipilimumab and nivolumab were reported, with response rates up to 44% [35,36]. In one of those studies, long lasting responses were explicitly described in patients with pancreatic NET G3 [36].

Finally, highly selected patients receiving individualized multimodal treatment approaches were amongst the longest responders, emphasizing the need to discuss NET G3 cases in a multidisciplinary setting.

In summary, in first-line treatment, the highest ORR could be observed in patients receiving FOLFOX, the longest PFS in patients receiving TEM/CAP. In second-line, FOLFOX was the most effective regiment regarding ORR and PFS.

Our study has several limitations, the main one lying in its retrospective nature. Since the different first-line treatment groups were not randomized, observed differences could be caused by differences in the patient characteristics rather than in the applied treatments. However, analysis for risk factors of PFS only showed that the choice of PE vs. non-PE treatment was significant at a univariate level. Patient numbers in several treatment groups, especially in second-line, were quite low, making it necessary to subsume different therapeutic regimens into one heterogeneous group for the analysis. Furthermore, a central pathologic review of the diagnostic samples was not performed, meaning that potential low grade NEC patients could have been included into the study. However, baseline patient characteristics are in line with NET G3 characteristics published by other groups, and all pathological findings were carefully reviewed by investigators with long standing experience in diagnosis and treatment of NET in order to comply with the most current WHO classification. Furthermore, a recent reanalysis of the NORDIC NEC study showed a very similar biological behavior, especially regarding response and PFS to PE first-line therapy, of NET G3 and NEC with a Ki67 < 55% (named low-grade NEC), in contrast to NEC with a Ki67 > 55% [5]. Those findings challenge the current WHO classification defining the distinction between NET G3 and NEC on a morphological differentiation level, and not by Ki67. It can be assumed that an accidental inclusion of single low-grade NEC patients in our study should not have a substantial impact on the observed outcomes, at least regarding PE treatment. Furthermore, a central radiology review was not performed in our study. Additionally, our analysis of treatment data did not contain toxicity data for the different treatments applied. However, valid toxicity data are difficult to obtain in a retrospective multi-center study with multiple heterogeneous treatments, with no prespecified patient visits, and is highly suffering from reporting bias. Furthermore, all regimens reported in the analysis are well established therapies in NETs and a multitude of other tumor entities and have a well-known toxicity profile drawn by analysis of multitude of patients treated in a prospective setting, also including randomized and blinded trials. Finally, we did not report OS data for the different treatment arms since general OS data of the whole cohort were not mature, and there was a great heterogeneity in applied treatments in comparatively small groups, with many patients receiving multiple lines. Despite these limitations, our analysis reflects the current therapeutic strategy and the treatment outcomes for NET G3 in a real-world setting.

## 5. Conclusions

In summary, this study is the largest analysis of NET G3 up to date, and the first one comparing different first-line regimens for this recently defined entity. The observed data reflect the current treatment strategy and can serve as a basis for further prospective studies, either testing established treatment options in a randomized setting or evaluating novel investigative therapeutic approaches. There is an unmet medical need for further trials defining the optimal medical management of this newly defined tumor entity, taking different primary sites and molecular features into consideration.

## Figures and Tables

**Table 1:** Patient characteristics.

Characteristics			Number of Patients (*n* = 142)	%
Age (years)	Median (Range)	57 (14–81)		
Sex	Male		79	55.6
	Female		63	44.4
Stage	I		2	1.4
	II		7	4.9
	III		26	18.3
	IV		107	75.4
Ki67 (%)	Median (Range)	30 (21–70)		
Primary	Pancreas		99	69.7
	Small intestine		13	9.2
	Stomach/esophagus		8	5.6
	Colorectal		3	2.1
	Other		3	2.1
	Unknown		16	11.3
SSTR Imaging	Positive		64	45.1
	Negative		19	13.4
Prior NET G1/G2			23	16.2
Functional activity			16	11.3
Metastatic sites	Median (Range)	2 (0–4)		
	Liver		123	86.6
	Lymph nodes		70	49.3
	Bone		34	23.9
	Lung		14	9.9
	Peritoneum		19	13.4
	Brain		4	2.8
	Pleura		2	1.4
	Other		14	9.9
	Liver only		41	28.9
LDH	Median [U/mL] (Range)	249.0 (110.0–1647.0)		
	>ULN		16	11.3
	≤ULN		87	72.5
CgA	Median [ng/mL] (Range)	256.6 (5.0–220,900.0)		
	>ULN		101	71.1
	≤ULN		20	14.1
NSE	Median [ng/mL] (Range)	29.05 (5.2–346.9)		
	>ULN		96	67.6
	≤ULN		12	8.5

CgA: chromogranin A; LDH: lactate dehydrogenase; NSE: neuron-specific enolase; SSTR: somatostatin receptor; ULN: upper limit normal.

**Table 2:** Response rates in different first-line treatment groups.

	PE	(%)	FOLFOX	(%)	TEM/CAP	(%)	STZ/5-FU	(%)	Other	(%)	*p*
**CR**	1	2.7	0	0.0	0	0.0	0	0.0	1	5.6	
**PR**	12	32.4	22	56.4	6	27.3	9	45.0	2	11.1	
**SD**	11	29.7	10	25.6	9	40.9	5	25.0	8	44.4	
**PD**	12	32.4	4	10.3	6	27.3	6	30.0	5	27.8	
**NE**	1	2.7	3	7.7	1	4.5	0	0.0	2	11.1	
**ORR**	13	35.1	22	56.4	6	27.3	9	45.0	3	16.7	0.032
*p* *		0.063		ref		0.028		0.406		0.005	
**DCR**	24	64.9	32	82.1	15	68.2	14	70.0	11	61.1	0.420

CR: complete response; DCR (CR + PR + SD): disease control rate; NE: not evaluable; ORR (CR + PR): overall response rate; *p* *: pairwise *p* value; PD: progressive disease; PR: partial response; ref: reference group for pairwise comparison; SD: stable disease.

**Table 3:** Patient characteristics in different first-line treatment subgroups.

Characteristics	PE (*n* = 37)	FOLFOX (*n* = 32)	TEM/CAP (*n* = 29)	STZ/5-FU (*n* = 20)	Other (*n* = 18)	*p*
Sex, male	19 (51.4%)	23 (59.0%)	14 (63.6%)	11 (55.0%)	9 (50.0%)	0.869
Age	59 (25–79)	62 (21–77)	60 (29–80)	50 (32–70)	51 (14–81)	0.045
Ki67	40 (21–70)	30 (22–70)	25 (21–50)	25 (20–60)	30 (21–70)	<0.001
*p* *	ref	0.728	0.004	<0.001	0.002	
*p* *	0.728	ref	0.002	<0.001	0.019	
Primary						0.044
Pancreas	22 (59.5%)	26 (66.7%)	16 (72.7%)	20 (100.0%)	10 (55.6%)	
Stomach/esophagus	3 (8.1%)	4 (10.3%)	0 (0.0%)	0 (0.0%)	1 (5.6%)	
Small intestine	1 (2.7%)	5 (12.8%)	2 (9.1%)	0 (0.0%)	5 (27.8%)	
Colorectal	2 (5.4%)	1 (2.6%)	0 (0.0%)	0 (0.0%)	0 (0.0%)	
Other	2 (5.4%)	0 (0.0%)	0 (0.0%)	0 (0.0%)	1 (5.6%)	
Unknown	7 (18.9%)	3 (7.7%)	4 (18.2%)	0 (0.0%)	1 (5.6%)	
SSTR positive	10 (62.5%)	15 (71.4%)	15 (78.9%)	12 (80.0%)	11 (100.0%)	0.228
Functional activity	1 (2.7%)	5 (12.8%)	1 (4.5%)	5 (25.0%)	4 (22.2%)	
Prior NET G1/G2	2 (5.4%)	12 (30.8%)	4 (18.2%)	2 (10.0%)	3 (16.7%)	0.049
Metastatic sites	1 (1–4)	2 (1–5)	2 (1–5)	2 (1–4)	3 (1–5)	0.018
*p* *	0.001	0.026	0.014	0.125	ref	
Liver only	16 (44.4%)	9 (23.7%)	6 (27.3%)	8 (40.0%)	1 (5.9%)	0.040
LDH						
Median [U/mL] (Range)	253.0 (3.9–495.0)	258.0 (168.0–1647.0)	247.0 (179.0–403.0)	200.5 (110.0–292.0)	231.0 (144.0–420.0)	0.192
>ULN	3 (10.0%)	9 (27.3%)	2 (12.5%)	0 (0.0%)	1 (6.7%)	0.201
≤ULN	27 (90.0%)	24 (72.7%)	14 (87.5%)	4 (100.0%)	14 (93.3%)	
CgA						
Median [ng/mL] (Range)	233.5 (16.3–220,900.0)	494.9 (5.0–84,240.0)	294.8 (60.3–42,260.0)	256.2 (37.4–1973.0)	293.1 (27.4–5867.0)	0.704
>ULN	28 (84.8%)	29 (80.6%)	16 (84.2%)	14 (93.3%)	11 (73.3%)	0.665
≤ULN	5 (15.2%)	7 (19.4%)	3 (15.8%)	1 (6.7%)	4 (26.7%)	
NSE						
Median [ng/mL] (Range)	29.1 (5.2–205.7)	36.7 (16.1–346.9)	31.2 (8.0–105.7)	29.2 (13.1–92.0)	24.5 (15.6–51.8)	0.166
>ULN	26 (86.7%)	33 (97.1%)	16 (88.9%)	6 (75.0%)	12 (80.0%)	0.280
≤ULN	4 (13.3%)	1 (2.9%)	2 (11.1%)	2 (25.0%)	3 (20.0%)	

*p* *: pairwise *p* value; ref: reference group for pairwise comparison.

**Table 4:** Progression-free survival of first-line therapy (univariate, multivariate analysis).

Variables	Univariate		Multivariate	
	HR (95% CI)	*p*	HR (95% CI)	*p*
Sex	1.262 (0.860–1.851)	0.234	1.214 (0.655–2.250)	0.537
Age *	1.001 (0.987–1.015)	0.893		
Primary pancreatic	0.987 (0.656–1.483)	0.948		
Ki67 *	1.007 (0.991–1.022)	0.392		
SSTR positive	0.681 (0.380–1.220)	0.194	0.728 (0.350–1.514)	0.395
Prior NET G1/G2	0.977 (0.588–1.624)	0.928		
Metastatic sites *	0.969 (0.807–1.163)	0.735		
Liver only	0.996 (0.656–1.512)	0.984		
LDH *	1.000 (0.999–1.002)	0.498		
LDH elevated	1.005 (0.510–1.980)	0.988		
CgA *	1.000 (1.000–1.000)	0.328		
CgA elevated	1.214 (0.696–2.118)	0.493		
NSE *	1.003 (0.999–1.006)	0.112	1.003 (0.998–1.008)	0.189
NSE elevated	1.118 (0.588–2.124)	0.734		
Non-PE vs. PE	0.666 (0.443–1.000)	0.049	0.773 (0.390–1.530)	0.459

* Continuous variables.

**Table 5:** Efficacy of second-line therapy.

	PE	%	FOLFOX	%	TEM/CAP	%	FOLFIRI	%	Everolimus	%	Other	%
**CR**	0	0.0	0	0.0	0	0.0	0	0.0	0	0.0	0	0.0
**PR**	6	46.2	4	40.0	4	20.0	1	9.1	2	16.7	6	18.2
**SD**	3	23.1	4	40.0	9	45.0	2	18.2	3	25.0	9	27.3
**PD**	4	30.8	1	10.0	4	20.0	5	45.5	6	50.0	15	45.5
**NE**	0	0.0	1	10.0	3	15.0	3	27.3	1	8.3	3	9.1
**ORR**	6	46.2	4	40.0	4	20.0	1	9.1	2	16.7	6	18.2
**DCR**	9	69.2	8	80.0	13	65.0	3	27.3	5	41.7	15	45.5
**PFS (months)**	8.0		13.9		7.7		2.4		4.0		4.2	

## Data Availability

The data presented in this study are available on request from the corresponding author. The data are not publicly available due to privacy and ethical reasons.

## Supplementary Materials

The following are available online at https://www.mdpi.com/article/10.3390/cancers13081936/s1. Table S1: Progression-free survival of first-line therapy, multivariate analysis for two covariates excluding multimodally treated patients. Table S2: Baseline characteristics of different treatment groups in second-line. Figure S1: Progression-free survival of different first-line regimens for non-pancreatic primary. Patients receiving SSAs, PRRT, other chemotherapeutic regimens, targeted agents and multimodal combination approaches were subsumed under other.
