# Supplementary material for: Multicenter Analysis of Treatment Outcomes for Systemic Therapy in Well Differentiated Grade 3 Neuroendocrine Tumors (NET G3)
Source: Cancers (Basel). 2021 Apr 16;13(8):1936. doi: 10.3390/cancers13081936 (PMC8073753; doi:10.3390/cancers13081936)
Supplement: Supplementary file 1 [file cancers-13-01936-s001.zip › cancers-1148986-supplementary.pdf]

cancers

MDPI

# Supplemental Materials: Multicenter Analysis of Treatment Outcomes for Systemic Therapy in Well Differentiated Grade 3 Neuroendocrine Tumors (NET G3)

**Leonidas Apostolidis, Arianna Dal Buono, Elettra Merola, Henning Jann, Dirk Jäger, Bertram Wiedenmann, Eva Caroline Winkler and Marianne Pavel**

**Supplementary table S1.** Progression-free survival of first-line therapy, multivariate analysis for two covariates excluding multimodally treated patients.

| | Multivariate | |
|---|---|---|
| | HR (95 % CI) | P |
| Ki67 * | 1.001 (0.985–1.107) | 0.944 |
| Non-PE vs. PE | 0.766 (0.500–1.175) | 0.222 |

* Continuous variables.

**Supplementary table S2.** Baseline characteristics of different treatment groups in second-line.

| | PE (*n* = 13) | FOLFOX (*n* = 10) | TEM/CAP (*n* = 20) | FOLFIRI (*n* = 11) | Everolimus (*n* = 12) | Other (*n* = 33) |
|---|---|---|---|---|---|---|
| Sex, male | 8 (61.4%) | 6 (60.0%) | 13 (65.0%) | 8 (72.7%) | 5 (41.7%) | 13 (39.4%) |
| Age | 50 (31–69) | 54 (37–81) | 58 (32–80) | 68 (33–75) | 48 (25–61) | 56 (14–79) |
| Ki67 | 30 (21–50) | 30 (25–60) | 30 (21–50) | 40 (30–70) | 28 (21–60) | 25 (20–70) |
| Primary | | | | | | |
| Pancreas | 8 (61.5%) | 5 (50.0%) | 16 (80.0%) | 6 (100.0%) | 10 (55.6%) | 22 (66.7%) |
| Stomach/esophagus | 0 (0.0%) | 2 (20.0%) | 1 (5.0%) | 0 (0.0%) | 1 (5.6%) | 3 (9.1%) |
| Small intestine | 0 (0.0%) | 3 (30.0%) | 0 (0.0%) | 2 (0.0%) | 5 (27.8%) | 4 (12.1%) |
| Colorectal | 0 (0.0%) | 0 (0.0%) | 0 (0.0%) | 1 (0.0%) | 0 (0.0 %) | 1 (3.0%) |
| Other | 1 (7.7%) | 0 (0.0%) | 0 (0.0%) | 1 (0.0%) | 1 (5.6%) | 1 (3.0%) |
| Unknown | 4 (30.8%) | 0 (0.0%) | 3 (15.0%) | 1 (0.0%) | 1 (5.6%) | 2 (6.1%) |
| SSTR positive | 5 (62.5%) | 4 (57.1%) | 4 (33.3%) | 5 (100.0%) | 8 (100.0%) | 19 (86.4%) |
| Functional activity | 0 (0.0%) | 2 (20.0%) | 3 (15.0%) | 2 (18.2%) | 2 (16.7%) | 4 (12.1%) |
| Prior NET G1/G2 | 0 (0.0%) | 4 (40.0%) | 1 (5.0 %) | 3 (27.3%) | 1 (8.3%) | 7 (21.2%) |
| Metastatic sites | 2 (1–4) | 2 (1–5) | 2 (1–4) | 2 (1–5) | 1 (1–3) | 2 (1–5) |
| Liver only | 1 (7.7%) | 4 (40.0%) | 4 (20.0 %) | 3 (27.3 %) | 1 (8.3%) | 7 (21.1%) |
| Best response to first-line | | | | | | |
| CR/PR | 5 (83.5%) | 3 (30.0%) | 7 (35.0%) | 6 (54.4%) | 6 (50.0%) | 13 (39.4%) |
| CR/PR/SD | 8 (61.5%) | 5 (50.0%) | 15 (75.0%) | 8 (72.7%) | 10 (83.3%) | 24 (73.7%) |
| PD | 5 (38.5 %) | 5 (50.0%) | 5 (25.0%) | 3 (27.3%) | 2 (16.7%) | 9 (27.3%) |

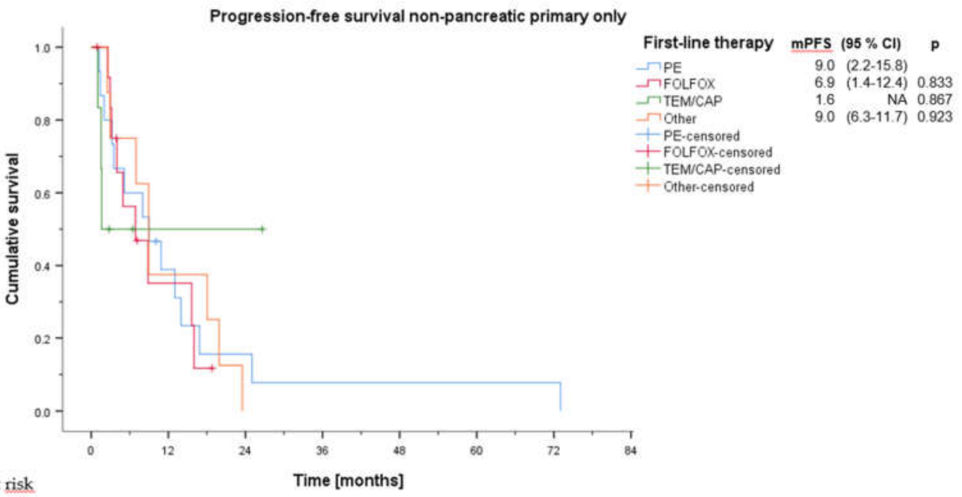


Patients at risk

| | | | | | | | | |
|---|---|---|---|---|---|---|---|---|
| PE | 15 | 5 | 2 | 1 | 1 | 1 | 1 | 1 |
| FOLFOX | 13 | 3 | 0 | 0 | 0 | 0 | 0 | 0 |
| TEM/CAP | 6 | 1 | 1 | 0 | 0 | 0 | 0 | 0 |
| Other | 8 | 3 | 0 | 0 | 0 | 0 | 0 | 0 |

(a)

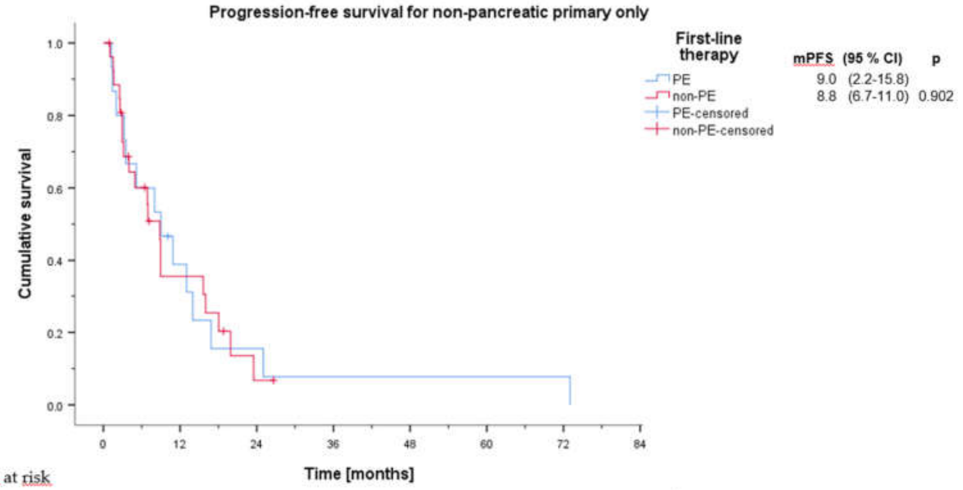


Patients at risk

| | | | | | | | | |
|---|---|---|---|---|---|---|---|---|
| PE | 15 | 5 | 2 | 1 | 1 | 1 | 1 | 1 |
| Non-PE | 27 | 7 | 1 | 0 | 0 | 0 | 0 | 0 |

(b)

**Supplementary figure S1.** Progression-free survival of different first-line regimens for non-pancreatic primary. Patients receiving SSAs, PRRT, other chemotherapeutic regimens, targeted agents and multimodal combination approaches were subsumed under other. All *p* values are delineated in comparison to platinum + etoposide (PE). NA: not available. (**a**) Comparison of the main treatment groups. (**b**) Comparison of PE vs. non-PE.
